# Nodules cutanés négligés révélant un adénocarcinome pulmonaire

**DOI:** 10.11604/pamj.2016.24.131.8686

**Published:** 2016-06-10

**Authors:** Iliass El Alami, Hassan Errihani

**Affiliations:** 1 Departments of Medical Oncology, Military Hospital Med V, Rabat, Morocco

**Keywords:** Nodules cutanés, adénocarcinome pulmonaire, tabagique chronique, SKin nodules, pulmonary adenocarcinoma, nicotine dependence

## Image en médecine

Nous rapportons le cas d'un patient de 45 ans, tabagique chronique à raison de 20 paquets année qui s'est présentait en consultation d'oncologie médicale pour des nodules sous cutanés multiples au niveau de l'abdomen associés à une lésion ulcérée de l'hypochondre droit mesurant (10x10cm) rapidement évolutive en moins de 2 mois. Une biopsie faite montrait une localisation secondaire d'un adénocarcinome dont l’étude immunohistochimique révèle l'origine broncho-pulmonaire (TTF1 et CK7 positifs). Le bilan d'extension par scanner thoraco-abdomino-pelvien objectivait un processus tumoral du poumon droit; ainsi une chimiothérapie palliative a été indiqué. Le cancer du poumon est souvent diagnostiquait à un stade métastatique, avec une prédilection à des localisations ganglionnaire, pleurale, pulmonaire controlatéral, cérébral, osseuse et surrénalienne par ailleurs les métastases cutanées sont rares avec une incidence de 2,9-5,3% pour tous les cancers et de 1-12% pour le cancer du poumon. Les lésions sont souvent faite d'un seul nodule à plusieurs sous cutanée dur immobile avec une taille variant de (0,5 à 10 cm), les métastases cutanées d'origine pulmonaire sont souvent de type adénocarcinome suivi du carcinome épidermoide ainsi que le carcinome pulmonaire à petites cellules, notre patient présentait une des métastases sous cutanées multiples dont une grosse s'est ulcérée.

**Figure 1:**
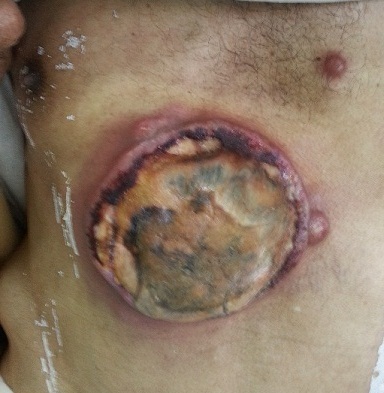
nodule cutané ulcéré

